# Inclusion of Older Adults in Digital Health Technologies to Support Hospital-to-Home Transitions: Secondary Analysis of a Rapid Review and Equity-Informed Recommendations

**DOI:** 10.2196/35925

**Published:** 2022-04-27

**Authors:** Kristina Marie Kokorelias, Michelle LA Nelson, Terence Tang, Carolyn Steele Gray, Moriah Ellen, Donna Plett, Carlotta Micaela Jarach, Jason Xin Nie, Kednapa Thavorn, Hardeep Singh

**Affiliations:** 1 St John's Rehab Research Program Sunnybrook Research Institute Sunnybrook Health Sciences Centre Toronto, ON Canada; 2 Department of Medicine Sinai Health System/University Health Network Toronto, ON Canada; 3 Lunenfeld-Tanenbaum Research Institute Sinai Health Toronto, ON Canada; 4 Institute of Health Policy, Management and Evaluation University of Toronto Toronto, ON Canada; 5 March of Dimes Canada Toronto, ON Canada; 6 Institute for Better Health Trillium Health Partners Toronto, ON Canada; 7 Department of Medicine University of Toronto Toronto, ON Canada; 8 Department of Health Policy and Management Ben-Gurion University of the Negev Eilat Israel; 9 Guilford Glazer Faculty of Business and Management Ben-Gurion University of the Negev Eilat Israel; 10 Faculty of Health Sciences Ben-Gurion University of the Negev Eilat Israel; 11 Department of Environmental Health Sciences Istituto di Ricerche Farmacologiche Mario Negri IRCCS Milan Italy; 12 Ottawa Hospital Research Institute School of Epidemiology and Public Health University of Ottawa Ottawa, ON Canada; 13 Department of Occupational Science & Occupational Therapy Temerty Faculty of Medicine University of Toronto Toronto, ON Canada

**Keywords:** older adults, digital technology, transitions, older adult population, digital health, Digital Hospital, health intervention, aging, gender diversity, home transition, epidemiology

## Abstract

**Background:**

Digital health technologies have been proposed to support hospital-to-home transition for older adults. The COVID-19 pandemic and the associated physical distancing guidelines have propelled a shift toward digital health technologies. However, the characteristics of older adults who participated in digital health research interventions to support hospital-to-home transitions remain unclear. This information is needed to assess whether current digital health interventions are generalizable to the needs of the broader older adult population.

**Objective:**

This rapid review of the existing literature aimed to identify the characteristics of the populations targeted by studies testing the implementation of digital health interventions designed to support hospital-to-home transitions, identify the characteristics of the samples included in studies testing digital health interventions used to support hospital-to-home transitions, and create recommendations for enhancing the diversity of samples within future hospital-to-home digital health interventions.

**Methods:**

A rapid review methodology based on scoping review guidelines by Arksey and O’Malley was developed. A search for peer-reviewed literature published between 2010 and 2021 on digital health solutions that support hospital-to-home transitions for older adults was conducted using MEDLINE, Embase, and CINAHL databases. The data were analyzed using descriptive statistics and qualitative content analysis. The Sex- and Gender-Based Analysis Plus lens theoretically guided the study design, analysis, and interpretation.

**Results:**

A total of 34 studies met the inclusion criteria. Our findings indicate that many groups of older adults were excluded from these interventions and remain understudied. Specifically, the *oldest old* and those living with cognitive impairments were excluded from the studies included in this review. In addition, very few studies have described the characteristics related to gender diversity, education, race, ethnicity, and culture. None of the studies commented on the sexual orientation of the participants.

**Conclusions:**

This is the first review, to our knowledge, that has mapped the literature focusing on the inclusion of older adults in digital hospital-to-home interventions. The findings suggest that the literature on digital health interventions tends to operationalize older adults as a homogenous group, ignoring the heterogeneity in older age definitions. Inconsistency in the literature surrounding the characteristics of the included participants suggests a need for further study to better understand how digital technologies to support hospital-to-home transitions can be inclusive.

## Introduction

### Background

Transitioning across health care settings is a complex experience for older adults and their caregivers [1,2]. Older adults [3] and family caregivers (ie, family members, friends, or neighbors) who provide unpaid assistance or care to someone living with an injury, disability, or illness [4] frequently experience unmet care needs as the patients leave the hospital and transition to home [5-10]. Transitions in care are often more difficult for older adults who experience frequent hospitalizations and are often discharged with ongoing and complex care needs exceeding those that existed at the initial hospitalization [11,12]. Thus, researchers have urged integrated care strategies to better meet their care needs after hospitalization [12]. Here, we define integrated care as “the promotion of the comprehensive delivery of quality services across the life-course, designed according to the multidimensional needs of the population and the individual and delivered by a coordinated multidisciplinary team of providers working across settings and levels of care” [13].

Unsupported hospital-to-home transitions can result in adverse events, such as medication-related problems (eg, harmful drug effects) [14], readmissions to hospitals [15], lack of continuity of care [16], and even mortality [17,18]. To help overcome challenges during this transition period, older adults and their family caregivers attempt to develop, integrate, and use knowledge and skills to manage transitions in care settings and related changes in illness trajectories [19]. Improving transitions in care can help improve the quality and cost of care and promote more equitable care for vulnerable older adults [20]. An emerging area of research is the use of technology to help support hospital-to-home transitions for patients and their family caregivers [2,20-22].

Technological advances may help integrate health and social care in at-risk populations [23]. Technologies aimed at improving health outcomes for older adult populations as they transition across care settings have demonstrated success and promise [20,24-28]. Technologies to support care transitions can increase access to support for older adults as they transition from hospital-to-home by reducing architectural and physical barriers to accessing care in the community [20,29,30]. Other benefits of technology in supporting care during transitions include eliminating barriers to attending in-person support programs, such as restricted mobility, time constraints, transportation costs, and a lack of respite care for individuals caring for others [31].

Spurred by the COVID-19 pandemic, as face-to-face care options became less available initially, health systems and providers turned to digital tools as an alternate means of supporting older adults and families [32-34]. During this *digital revolution* [35], there has been increasing attention to whether or how health technologies support equitable access and use for all older adults who may benefit [36,37]. The rapid virtualization of health and social care to support hospital-to-home transitions poses a risk to access and equity and may create structural inequalities [38].

Older adults may be most vulnerable to inequitable access to and use of digital health technologies, given their overall lack of use of existing technologies [39]. Barriers to using technology for older adults include lower levels of digital literacy, lack of perceived usefulness, and physical and cognitive deficits that may make using digital tools challenging [40]. Similarly, previous studies have shown that older adults are overlooked in technological health research [41,42]. Barriers to technology use are even more prevalent in older adults from racial or ethnic minorities and socioeconomically disadvantaged groups [43]. Therefore, an equity-informed review of existing programs is required to create equity-informed guidelines to guide future development, delivery, and implementation of technologies to support hospital-to-home transitions for older adults. In the context of human experiences, including experiences with transitions in case, experiences are shaped by multiple social positions [44,45]. Moreover, a *one-size-fits-all* approach to transitional interventions may not work well for all people, of all social identities, given the high adverse events during transitional periods among persons from minority groups (eg, racial minority groups [46] and nonheterosexual individuals living in poverty [47]). Researchers have a growing interest in examining intersectionality in qualitative and quantitative research [44]. By including both qualitative and quantitative research in our review and noting how well the characteristics of particular groups have been reported, we hope to provide direction for future studies to better examine the multiple social positions left out of digital transitional care intervention research. Despite growing awareness of digital inequity, there are current knowledge gaps related to intersectionality and transitions, particularly within digital health interventions [48]. Addressing these knowledge gaps is a priority for the digital bridge intervention currently being developed by our research team [2,49]. Moreover, our results will provide recommendations that will inform the design and structure of other future digital health interventions that support hospital-to-home transitions for older adults.

### Objectives

To help inform recommendations for future technologies to assist with hospital-to-home transitions for older adults, we conducted a secondary analysis of a rapid review of existing technologies. The protocol for this broader review has been published elsewhere [21]. The initial review mapped the published literature on studies that tested digital health interventions to support hospital-to-home transitions. This review included all relevant interventions with samples of at least one older adult for comprehensiveness. Preliminary findings from the review indicated that less than one-fifth of the included studies were conducted exclusively with older adults and highlighted the need to explicitly examine interventions with older adults [21]. The broader review did not consider sex nor gender in its analysis, nor any other intersectional factors that influence participation in digital technology interventions. A secondary analysis focusing on sex, gender, and other intersectional factors was not part of the planned protocol [21]. Thus, the purpose of this secondary analysis was to (1) identify the characteristics of older adults targeted by studies testing the implementation of digital health interventions to support hospital-to-home transitions; (2) identify the characteristics of the samples included within studies testing digital health interventions to support hospital-to-home transitions; and (3) create recommendations for enhancing equity, diversity, and inclusion in future digital health intervention research. The specific research questions for this secondary analysis were as follows: “What are the targeted populations within existing digital health interventions supporting hospital-to-home transitions?” “What are the actual participants within existing digital health interventions supporting hospital-to-home transitions?”

## Methods

### Design

A rapid review was deemed appropriate, given the need to generate timely recommendations for future digital health interventions, as the COVID-19 pandemic has prompted an immediate need for novel technological supports [21,50,51]. Consistent with prior studies that conducted a secondary analysis of reviews [52-54], a secondary analysis entailed reexamining relevant data to answer different research questions and addressing knowledge gaps identified in the initial review [55]. We used modified and hybrid guidelines for rapid reviews [56] and the systematic guidelines of Arksey and O’Malley for scoping reviews [57,58]. This approach was deemed appropriate because scoping reviews allow for an iterative approach to data collection and analysis, whereas rapid reviews allow a timely synthesis of the existing literature. For example, we limited the search to select databases and conducted this review in a short period [59]. Our 5-stage rapid scoping review model included (1) identifying the research question, (2) identifying relevant studies, (3) selecting studies, (4) charting data, and (5) summarizing and reporting the results [58]. In the remainder of this section, we outline the specific steps undertaken to complete the review. As this secondary analysis aimed to answer different research questions than intended within the published protocol, the methods used in this study necessitated some deviations from the original protocol, as described in the following sections [21].

As there are no reporting guidelines for rapid reviews, we relied on elements of the PRISMA (Preferred Reporting Items for Systematic Reviews and Meta-Analyses) Protocols checklist as a guide for reporting this review [60].

### Theoretical Framework

This study was theoretically informed by a Sex- and Gender-Based Analysis Plus (SGBA+) lens [61]. The SGBA+ lens has been applied in the context of other reviews in health research [62,63]. As a theoretical framework, SGBA+ draws on intersectionality frameworks. Other intersectional frameworks include the Theoretical Domains Framework [64] and intersectionality-based policy analysis framework [65]. However, SGBA+ was specifically chosen, as it allowed researchers to examine sample characteristics within research processes and data, including biological sex and the multiple social positions that older adults hold (eg, ethnicity, income, age, race, education, and gender) to determine whether intervention findings are relevant to the needs of all older adults [61,66]. For this review, sex is defined here as a biological construct. In contrast, gender is defined as a social construct that refers to the socially prescribed dimensions of being a *female* or *male* [67].

This review explores how existing digital health interventions supporting hospital-to-home transitions represent sex, gender, and identity perspectives within their target and actual samples. These insights can be used to create equity-informed recommendations for future digital health interventions.

### Identifying the Research Question

The widespread shift to digital health during the COVID-19 pandemic has revealed digital equity to be a critical issue [38]. During the analysis phase of the larger rapid review [21], we identified the need to re-examine the data for identification.

### Identifying Relevant Studies

Relevant literature on digital health solutions currently applied to facilitate the transition from hospital-to-home for older adults was searched for as part of a larger review. A comprehensive, peer-reviewed search was created by an experienced information specialist in consultation with the research team and translated by the information specialist to MEDLINE (Ovid), CINAHL, and Embase (Ovid). The search was run on these databases by HC on November 26, 2020, for the larger review. In addition, the reference lists of 20 included articles were examined, and 6 content experts were consulted to identify additional studies for the larger review.

For this analysis, KMK and HS reran this search on September 20, 2021, using established guidelines [68] to ensure articles are up-to-date. KMK and HS used the same search strategy reported in the published protocol, including concepts related to *digital health*, *navigation*, and *transition of care from hospital to home* [21]. New (unique) articles retrieved from the updated search were reviewed as described in the following sections.

### Selecting Studies

Studies were included in the larger review [21] if they (1) empirically tested a digital health intervention and (2) supported a hospital-to-home transition (ie, continued from the hospital-to-home or community settings). The intervention had to be (3) tested with older adults (aged ≥65 years) who were recruited before their hospital discharge, (4) conducted in high-income countries [69], and (5) published in English in or after the year 2010 [21]. No limitations were imposed on the study design. The larger review was limited to interventions conducted in high-income countries for two reasons: digital and health infrastructure and resources can differ between high- and low-income countries, and the intent of the primary review was to provide recommendations for the digital bridge (a digital health intervention currently under development) [21,70]. As per the protocol, studies were excluded if the hospital setting was ambulatory (eg, emergency department visits) or if the discharge destination was an institution (eg, long-term care) [21]. We deviated from the protocol by limiting this review to technological interventions that are not strictly telephone based, given the extensive investigations and syntheses of telephone-based health interventions [71-75]. We also reduced the age of older adults to ≥55 years to be comprehensive to ensure *young old* adults are included [76].

As per the published protocol [21], study selection within the larger review used a single screener strategy after minimum interrater reliability was achieved (κ=0.80) during the title and abstract screening phases (ie, reviewed titles and abstracts together). Owing to the complexity of the inclusion criteria and limited information in titles and abstracts, we only screened for inclusion criteria 1, 4, and 5 during the title and abstract screening, whereas the remaining were screened for full-text review [21]. Interrater reliability was not reexamined during the full-text review stage, as we decided that 2 reviewers (KMK and HS) would independently screen articles at this stage because the papers had already undergone rigorous screening and interrater calculations. This secondary analysis did not need to be screened, as the purpose was to conduct an additional analysis to explore a question not addressed in the original study.

The study selection for this secondary analysis was modified from the published protocol to enhance comprehensiveness. The first author (KMK) independently reviewed the titles and abstracts of articles excluded from the larger review on August 31, 2021, to ensure that no potential article was missed with the single screener approach. However, no additional relevant articles were identified. After the search was updated for this review, 4 authors (KMK, DP, CMJ, and HS) reviewed the titles and abstracts (ie, 2 reviewers independently screened each article) over a 3-week period. After screening all titles and abstracts, 2 individuals (KMK and HS) reviewed articles from the initial full-text review and the updated search over an additional 3-week period. Team discussions, led by the senior author (HS), were used to resolve conflicts for both searches (ie, discrepancies in inclusion and exclusion and reasons for exclusion) until 100% agreement was obtained. Covidence software was used to facilitate the screening process [77].

### Charting the Data

The first author extracted data from the included articles using a modified form from the larger study. Extracted data included the study characteristics (ie, author, year, country, and design), details of the study inclusion criteria (ie, target sample), and details of the participants (ie, actual sample). Next, a spreadsheet was used to categorize the studies into three categories informed by SGBA+: sex, gender, and other identity constructs. All extracted data were reviewed and verified by a second reviewer (HS) to enhance the data quality and accuracy. Data were collected over approximately 2 months.

### Summarizing and Reporting the Data

Data were organized numerically using descriptive statistics and summarized using a narrative descriptive synthesis [78]. The narrative descriptive synthesis entailed the first and senior author mapping the findings into deductive themes informed by the SGBA+ framework, including sex, gender, geography, culture, age, and disability [61,66]. After coding all studies, the data were classified into 9 broad identity constructs. The constructs represented in this review included age, patient population, race and ethnicity, sex and gender, sexual orientation, education, disability, language, and technology access and comfort.

## Results

### Overview

**Figure 1:**
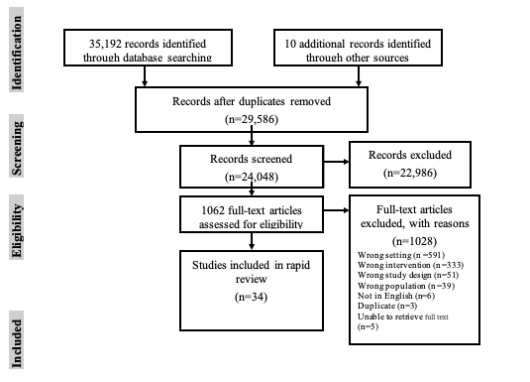
PRISMA (Preferred Reporting Items for Systematic Reviews and Meta-Analyses) diagram adapted from Moher et al [60].

In total, 34 articles met the inclusion and exclusion criteria. The search process is outlined in Figure 1. A total of 16 studies were conducted in Europe [79-94], 12 were conducted in North America [3,73,95-105], 3 in Asia [106-108], and 2 in Australia [109,110]. Multimedia Appendix 1 shows the distribution of studies based on location. In addition, of 34 studies, 1 (n=1, 3%) study used qualitative methodology [111], 1 (n=1, 3%) study was a report [98], and another used a case study design (n=1, 3%) [81]. A total of 9% (n=3) of studies used a mixed methods methodology [3,87,96], whereas the remaining studies (28/34, 82%) used a quantitative methodological approach. Of the 28 quantitative studies, 8 (n=8, 28%) used a randomized controlled trial design [89,90,97,101,106,107,109,110]. Other quantitative studies have used observational or nonrandomized trial designs.

Across all studies, 9809 participants were included (mean 297 participants per study, range 1 [65] to 3661 [70], SD 383). Across the 8 randomized controlled trials, 4434 participants were included (sample size mean 986 per study).

A total of 7 studies reported smaller sample sizes because of particular inclusion and exclusion criteria and limitations of the interventions (eg, dropouts) [3,87,90,91,100,102,105]. However, a small sample size was a deliberate choice for scholars in 2 studies [3,100].

### Digital Health Interventions

#### Overview

Multimedia Appendix 2 [3,83-94,100-103,105-108,110,111] summarizes the breadth of the methodological characteristics, aims of the studies, and a brief description of the digital interventions in detail. Briefly, web-based, tablet, and mobile app tools are the most common means of delivering digital interventions [3,83-94,100-103,105-108,110,111]. Electronic health records and databases [79,81,95,97,98,102,104] have been widely used for digital innovation. The use of wearable body sensors or devices [80,99,107], web-based chatting platforms [82], and automated emails [109] were less common.

The focus of digital health interventions varied. For example, some were related to medication reconciliation [79,81,97,104], whereas others aimed at providing education (eg, about rehabilitative exercises), internet-based care, and resources [83,86,89,92,94,96,100,104-106,111] and improved communication and care coordination with older adults’ care providers [3,82,91,104,108]. A total of 2 interventions aimed to improve communication processes among health care providers regarding discharge processes and care plans [93,109]. Many interventions aimed at monitoring bodily function and health status (including mental health) [80,84,85,88,90,94,99,101-103,107,110], often to alert members of the older adult’s care team of the need to schedule follow-up appointments or calls to help prevent adverse effects [97,98]. One study used digital technology to support home-delivered meals [87].

Regarding the targeted samples in the studies, the minimum age for inclusion in 3 studies was 55 years [3,99,105]. Other studies required participants to have a minimum age of 60 to 65 years, except for one that used 70 [94] and 75 years [79]. Conversely, 2 studies had a maximum age of 75 [79,106] and 80 years [89]. Justifications for maximum ages were not provided. A total of 7 studies did not report on their targeted age but instead referred to geriatric patients [81,83,101,102,109,110] or “elders” [98]. Multimedia Appendix 3 [3,83-94,100-103,105-108,110,111] outlines the targeted populations of the included studies. It is worth noting that none of these studies specifically set out to include an analysis of heterogeneous groups of patients.

There was heterogeneity in the mean age of the participants included in the studies. The mean of age included older adult participants ranged from 65 to 69 years [83,99,105,111], 70 to 74 years [3,80,84,85,89,94,101,106,108], and 75 to 79 years [82,87,92,97,101] to 80 to 84 [86,88,90-93,96,104,107,109,110]. Only 2 studies had a mean age of ≥85 years [79,109]. A few studies did not specify the mean patient age [88,95,98,100,102,112].

The patient populations in all the studies included mainly frail geriatric patients or older adults. Only one study purposely examined older adults with cognitive impairment (ie, patients with mild cognitive impairment) and vascular cognitive impairment (eg, vascular dementia) [89]. In terms of their targeted population, many studies (n=14, 41%) excluded older adults with cognitive impairments [82, 84-86, 88-90, 92, 94, 101, 102, 105, 110, 111]. These studies excluded older adults who could not communicate because of cognitive challenges [107], postoperative delirium [112], and dementia [82,86,88,89,107].

Owing to the nature of our inclusion criteria, all patients were hospitalized, although the reasons for hospitalization varied. Hospitalizations included patients identified with nutritional risk (n=1, 3%) [87], chronic obstructive pulmonary disease (n=1) [88], heart failure (n=4) [88,90,94,101], diabetes (n=2) [81,105], and stroke (n=2) [3,83]. Two studies required participants to live with multimorbidity, defined as living with ≥2 chronic conditions [3,107]. One study included patients hospitalized for any nonelective reason [104]. A total of 14 studies included patients who underwent or had been scheduled for a surgical procedure [102], such as elective surgery [80], hip surgeries [82,86,92,96,108,111], total knee arthroplasty [106], oncological surgeries [84,85,100] (eg, lung or gastrointestinal cancers) [91], or cardiac or major vascular surgery [112]. The family caregivers of patients participated in 5 studies [96,100,102,107,110].

#### Racial, Ethnic, and Cultural Diversity in Digital Health Transition Interventions

Racial, ethnic, cultural, and religious diversity were rarely considered in the inclusion criteria or target sample.

A total of 23% (8/34) of studies described their actual sample’s ethnicity, race, and culture [85,96,100,101,104,105,111,112]. The samples within all these studies were primarily White, except one, which included participants who were primarily Black (75% of the sample) [105]. This study also included 1 Asian participant (5%) [105]. In contrast, one of the studies dichotomized participants’ race and ethnicity as *White* or *others* [100]. Participants were racially diverse in a study conducted by Choi et al [111], whereby participants were White (60%), African American (20%), Asian or Pacific Islander (7%), and Hispanic (13%). Similarly, in a study by Madigan et al [101], most of the sample was White, and the minority was African American (26%) [101]. Another study included participants who were White (68%), Hispanic (13%), Black (13%), and Asian (7%) [104]. Similarly, another study included African (15%) and Asian (4%) participants [112]. Multimedia Appendix 4 [3,83-94,100-103,105-108,110,111] describes the details of the participants (ie, actual sample). It is worth noting that none of these studies specifically set out to include an analysis of heterogeneous groups of patients.

#### Sex and Gender Diversity of Digital Health Transition Interventions

None of the articles aimed to recruit a specific sex or gender in their inclusion criteria or had sampled for both sex *and* gender diversity.

In their actual samples, the percentage of females (sex) in the studies ranged from 0% [81] to 100% [106]. All but 3 studies (n=31, 91%) [93,98,102] reported the sex of the included participants. One study had only females in the study [106]. One case study included only 1 male participant [81]. Most studies had almost equal proportions of males and females, with approximately 50% in each category [3,88,95,97,100] or proportions of sexes ranging between approximately 40% and 59% [79,80,91,99,105,107,111]. Most of the other studies had much higher (ie, ≥60%) proportion of females than males within the sample (n=15, 44%) [82, 86, 87, 90, 92, 96, 99, 101, 103, 104, 107-111]. A total of 18% (6/34) of studies had a higher proportion (≥60%) of males compared with females within the sample [80,81,84,85,89,94]. None of the studies reported on participants’ gender identities or representations of gender-diverse older adults.

#### Sexual Orientation

Sexual orientation was not reported in the inclusion criteria or the sample of any of the included studies.

#### Education

Education level or literacy was a requirement for participation in 3 studies. One had limited inclusion to “those with junior high school-level education or higher” [108], and the others had limited inclusion to “school attendance >3 years” [89] and “low-literate older adults” [111].

A total of 8 studies reported the educational level of the sample [85,87,89,96,100,105,111,112]. Of these studies, 2 reported the length of education (between an average of 8-10 years [87,89]), but they did not report the educational details (eg, level and type of education). Of the remaining studies, 5 primarily included participants with an educational level of high school or less [85,96,105,111,112]. Participants with predominantly higher-level education, such as college, university, or graduate training, have been reported in a few studies [96,100,105,111,112].

#### Disability

A few studies excluded older adults with sensory or communication impairments (eg, severe aphasia or hearing loss) to ensure their ability to use the technology [83-88, 102, 106-108, 111] and vision [84-86,88,89,102,106,111]. Studies have also excluded older adults with arthritis [106] and neurological disorders [106]. A total of 21% (7/34) of studies excluded older adults with life-threatening illnesses [86-88,92,99,104,107]. Having a good health status or efficient disease control was a requirement in some studies [101,106]. Older adults with psychological conditions (eg, depression) were excluded from some studies [82,83,88,89]. Older adults with stroke were excluded from 6% (2/34) of studies [86,89]. Finally, studies excluded older adults using a wheelchair [99], severe ambulatory impairment [84,85], or inability to walk independently with a gait aid [86].

#### Language

The participants’ language proficiency was not discussed in the actual sample. However, some studies identified language as an inclusion criterion, but the reasons were not specified. Specifically, English-speaking proficiency was required in 20% (7/34) of the studies [3,96,100,103,105,110,111]. Other language requirements included Dutch [84,85], Italian [89], Danish [86], and Swedish [83,88]. It is worth noting that these were the primary languages of the countries in which these studies were conducted.

#### Technology Access and Comfort

Although some studies required participants or a caregiver to have internet access in their home [84,85,92] or working telephone line [101,102,108,110], access to the internet or device was not a requirement in all studies [86]. For example, Backman et al [96] provided participants with a loaner device if they did not have access to a mobile phone or computer. Similarly, because of low recruitment, the inclusion criteria were broadened in 2 studies to include those who did not have a phone [84,85].

Some studies included those with low technical literacy, providing training on device use and assistance with device setup [80,86,96,103,107,111]. However, others require participants to have technical literacy, including the capability to use [84,85,87,107] or familiarity with the tested device [82,106].

## Discussion

### Principal Findings

To our knowledge, this is the first rapid review to synthesize the characteristics of older adults (aged ≥55 years) within digital health interventions supporting hospital-to-home transition using an equity lens. Specifically, we described the target and actual sample characteristics of the 34 studies. Our findings indicate that many older adults were not recruited within these interventions and remain understudied (eg, older adults with cognitive impairment and oldest older adults). This study relied on an intersectionality framework to understand how different social identities influence participation in digital health interventions to improve hospital-to-home transitions and, in turn, the digital divide. On the basis of the study findings, we created a list of research implications to enhance the consideration of equity variables to ensure meaningful participation for diverse groups of older adults within the target and actual samples of digital health interventions (Multimedia Appendix 5).

We noted variability across studies in the age groups of older adults who were targeted and, in turn, who were included in the studies. It is well known that the hospitalization experiences and subsequent health and social service needs of older adults differ significantly depending on age [113-116]. Some studies did not specify a target age group of older adults [81,83,98,101,102,109,110] and recruited participants based on setting or program (eg, aged acute ward [109] and geriatric ward [102]). However, others were limited to a maximum age of 80 years [89]. However, justification within studies limiting the maximum age was poor.

The theorization of *fourth age* typically starts around age 80 years (when studies cut older adults off) and is seen as a time of dependence in which additional care needs may be needed [115], which inevitably translates into differing needs among older adults and requires important consideration for future intervention development. Thus, we used an equity-informed lens to identify older adults aged >80 years as an understudied group. Others have also noted this gap in the literature; thus, older adults aged >80 years should be considered in future digital health interventions [117].

In addition to age, 2 studies required older adults to have a *good health* status because of the perceived ability of the researcher to use technology [83,102]. Many studies have excluded older adults with cognitive and functional impairments or a poor health status. Older adults with poor health status have worse outcomes during transitions in care than the general older adult population [118]. Thus, excluding older adults with a poor health status may result in greater health inequities [48]. Furthermore, this limits the transferability of evidence to practice, given the high number of older adults with dementia and other comorbidities requiring hospitalization and returning home [119]. An equity perspective taken by our review elucidates the need for future research to consider how interventions can be designed for or adapted to understudied groups of non–English-speaking older adults with poor health from racial and ethnic minority groups [120], as these groups may be most vulnerable to adverse events during hospital-to-home transitions [120-123].

In addition, many studies have limited their interventions to older adults with access to and comfort with technology. This criterion runs the risk that novel technologies to support hospital-to-home transitions are exclusionary rather than inclusive of the older adults they aim to help. Older adults often face numerous barriers to the effective use of technological interventions because of a lack of access to and experience and skills with digital tools [124,125]. In addition, older adults with lower socioeconomic status have reduced access to digital resources and may be unable to afford the technology or internet required to use digital tools [126]. Socioeconomic status affects digital access and health status [127]. Such interventions may cause or worsen access disparities, as specific groups of patients are known to fall behind the average population in terms of their use of virtual services (this is often referred to as the *digital divide*) [128]. Some of the included studies posited suggestions for recruiting individuals from lower socioeconomic status, including the provision of a loaner device that had data (providing internet access) to mitigate the reliance on a personal device or internet access and financial barriers [84,85,96]. Other studies included those with low technology comfort by providing training on device use and assistance with device setup [80,86,96,103,107,111]. However, some studies have excluded older adults with impaired sensory, cognitive, or communication functions. As these impairments are common in the oldest older adults [129], commonly referred to as the *oldest old* or *old old* (ie, ≥85 years) [130], this restriction may explain why studies tended to include those younger within the older adult category. Although these impairments could reduce participants’ ability to use digital intervention, their participation can be supported by adapting technologies that are compatible for people with disabilities to use [131]. Thus, hospital-to-home interventions seeking to incorporate digital technologies should consider the intersection between disability and age and offer training and practice for the implemented technology [132]. Future research should explore ways to meet the needs of older adults with various impairments by designing technology that is as inclusive as possible [133]. In efforts to reduce inequities related to age and disability, strategies such as including individuals with disabilities (eg, dementia [134]) in technological development have been used [135].

The digital divide (ie, the disadvantage of those who are either unable or do not choose to use technologies) is the largest among older adults with low education, older adults with limited English proficiency, and certain racial or ethnic groups (eg, Hispanic or Black) [136,137]. Simultaneously, there are also cohorts of older adults that commonly face health inequities in low-income countries [138,139]. Many of the studies included in this review did not report the minority languages or race and ethnicity of the sample. Systematic reviews have noted inequalities and disparities in access to various health services among racial, ethnic, and language minorities [140,141]. To help overcome barriers to care for minority populations, reliable reporting of such characteristics is necessary to target improvement efforts to ensure equitable access to care [142]. Future studies should report on racial, ethnic, and cultural backgrounds and experiences to ensure that the needs and experiences of these groups are considered [143]. Moreover, future studies should include strategies for recruiting diverse groups of participants by offering technologies in different languages [144]; using racially, ethnically, and culturally diverse research staff [145,146]; and providing compensation for participation [146]. Carefully worded recruitment advertisements can also support gender diversity within these groups [147]. Highlighting the various genders incorporated into current interventions can help make research recommendations for including more diversity in future interventions and studying sex- and gender-based differences.

### Limitations

In this secondary review of 34 articles describing the inclusion of older adult participants in hospital-to-home interventions, we experienced some limitations. First, our findings are limited to the data reported in the studies, and not all studies have reported particular characteristics (eg, education, race). Another limitation of our review is that we only included a synthesis of data that pertained to the SGBA+ framework and may have inadvertently excluded commentary on other meaningful measures of diversity (eg, immigration status). Second, we only included a synthesis of data that pertained to the SGBA+ framework and may have inadvertently excluded commentary on other meaningful measures of diversity (eg, immigration status). Third, our review was also limited by its rapid review methodology, whereby only one person screened the titles and abstracts in the larger review. In addition, we may have missed potentially relevant articles because of our use of a rapid methodology and searching for a limited number of databases. Fourth, there is a risk that articles may have been missed because of our search strategy, as digital health interventions are not described consistently [21]. However, it is worth noting that the intent of that study was not to capture all articles but to provide an overview of the literature [21]. Fifth, the results should be interpreted with caution, as we could not confidently determine which studies reported unique interventions versus the reported results of one intervention within multiple studies. Finally, we recommend that future studies examine digital health interventions in low- and middle-income countries, as our review is limited to digital health interventions in high-income countries.

### Conclusions

To the best of our knowledge, this is the first review that has mapped the literature focusing on the characteristics of older adults included in studies of digital interventions supporting hospital-to-home transition. These findings suggest that the literature on digital health interventions tends to operationalize older adults as a homogenous group, ignoring the heterogeneity in older age definitions. In addition, few studies have reported on racial, ethnic, cultural, or gender diversity, which can facilitate a further digital divide among older adults. Inconsistency in the literature surrounding the characteristics of the included participants suggests a need for further study to better understand how digital technologies to support hospital-to-home transitions can be inclusive. Specifically, the SBGA+ framework can inform future research and interventions to support older adults during hospital-to-home transitions.

## Appendix

Multimedia Appendix 1 Geographical spread of studies.

Multimedia Appendix 2 Supplementary table of study characteristics.

Multimedia Appendix 3 Targeted populations of the included studies.

Multimedia Appendix 4 Details of the participants (ie, actual sample).

Multimedia Appendix 5 List of research implications.

## Abbreviations

PRISMA: Preferred Reporting Items for Systematic Reviews and Meta-Analyses
SGBA+: Sex- and Gender-Based Analysis Plus
