# Supplementary figures and images for: Inclusion of Older Adults in Digital Health Technologies to Support Hospital-to-Home Transitions: Secondary Analysis of a Rapid Review and Equity-Informed Recommendations
Source: JMIR Aging. 2022 Apr 27;5(2):e35925. doi: 10.2196/35925 (PMC9096639; doi:10.2196/35925)

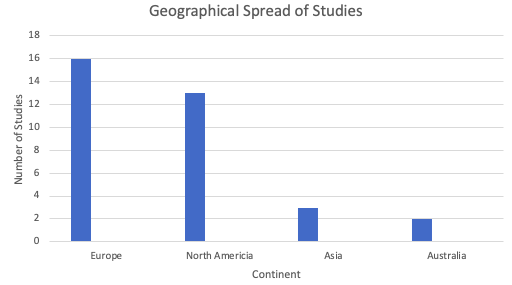

Supplement: Multimedia Appendix 1 [file aging_v5i2e35925_app1.png]
