# Supplementary material for: Inclusion of Older Adults in Digital Health Technologies to Support Hospital-to-Home Transitions: Secondary Analysis of a Rapid Review and Equity-Informed Recommendations
Source: JMIR Aging. 2022 Apr 27;5(2):e35925. doi: 10.2196/35925 (PMC9096639; doi:10.2196/35925)
Supplement: Multimedia Appendix 2 [file aging_v5i2e35925_app2.docx]

**Mutlimedia Appendix 2: Supplementary table of study characteristics**

**Table 1— Characteristics of Included Peer-Reviewed Studies**

| **First Author, Year, Country & Design** | **Objective** | **Brief description of the intervention** | |
| --- | --- | --- | --- |
| Agud et al. (2016)  Spain  Quantitative | To assess and analyze the results of medication reconciliation and information programme for discharge of geriatric patients conducted through hospital information systems | Medication reconciliation and information programme created using the Access® database and available to primary care physicians through Horus® visor information system | |
| Amroze et al. (2019)  United States  Quantitative | To evaluate the feasibility and effectiveness of the electronic health record (EHR) access and audit logs, to track primary care physicians’ (PCPs’) response and opening of the non-interruptive alerts that are delivered to the Electronic Health Record (EHR) InBasket EHR–based intervention to highlight postdischarge medication concerns using locally developed non-interruptive alerts deliver to EHR In Baskets | EHR–based intervention to highlight postdischarge medication concerns using locally developed non-interruptive alerts deliver to EHR In Baskets | |
| An et al. (2021)  Korea  Quantitative | To evaluate the effectiveness of preoperative telerehabilitation program by measuring outcomes, including muscle strength, knee range of motion, and pain, activities of daily living and quality of life among older adults scheduled for bilateral total knee arthroplasty (TKA) | The intervention contained a preoperative and postoperative intervention. Preoperative telerehabilitation intervention was a 3-week intensive exercise program (30 min/session, 2 times/day, 5 days/week), conducted 3 weeks before the TKA procedure, comprised of warm-up, mobility, flexibility, strength, balance and cool-down exercises. These interventions were performed at home using a video call on a device under the supervision of a therapist. The postoperative patient education group consisted of an education session about home exercise safety and the protocol of intervention details for 40 min and thereafter performed a self-home exercise. A non-supervised intervention was performed for 30 min per session, 2 times/day, 5 days/week for 3 weeks. Furthermore, a physical therapist also checked physical condition and provided daily notification, motivation and education of the preoperative exercise via telephone calls once every day | |
| Aziz et al. (2011)  United Kingdom  Quantitative | To determine the effectiveness and feasibility of the ear-worn activity recognition (e-AR) device to monitor patients’ activity levels during postoperative recovery in an unsupervised home setting | e-AR device (wireless ear-worn body sensor network device) for remote activity monitoring |  |
| Backman et al. (2020)  Canada  Mixed methods study | To develop and assess a new web-based application, *MyPath to Home,* as a tool to assist geriatric rehabilitation patients with transition from hospital to home | Patient - and clinician - tailored web-based application called *MyPath to Home* used to share/deliver discharge-related information and resources |  |
| Boeni et al. (2015)  Switzerland  Case study | To determine the effectiveness of a pharmaceutical care service combined with an electronic polypharmacy adherence monitoring, by reporting on a case study of a 65-year-old patient with diabetes after hospital discharge | Polypharmacy adherence monitoring merged into a pharmaceutical care service (counselling, electronic multidrug punch cards, feedback on recent electronic records) |  |
| Chen et al. (2010)  Australia  Quantitative | To analyze the practicability of delivering computer-generated discharge summaries to primary care settings by email, fax, post, and patient hand delivery for older patients | Electronic discharge summary sent to primary care by email, fax, post or patient hand delivery |  |
| Choi et al. (2016)  United States  Qualitative | To develop a new website with pictograph-formatted discharge instructions while assessing to enhance the relevance and clarity of the website based on evaluations from intended users | Website containing web-based, pictograph-formatted discharge instructions |  |
| Gao et al. (2021)  Spain  Quantitative | To understand the practicability and role of a communication platform, known as WeChat, to assist with the rehabilitation of older adults with hip fracture during the COVID-19 pandemic | WeChat was a communication platform in which doctors shared informative texts, images, voice and video messages with patients on a group chat to guide and encourage patients to perform the recommended rehabilitation exercises. Furthermore, personalized support was provided to some patients |  |
| Guidetti et al. (2020)  Sweden  Quantitative | To analyze the feasibility of an Information and Communication Technology intervention, known as F@ce for older adults with stroke | F@ce intervention, a person-centered and team-based, eight-week rehabilitation intervention that seeks to improve function based on principles of building a relationship with individuals with stroke, promoting sharing and encouraging for goal setting, to overall reduce the impact of stroke and help patients return to daily activities |  |
| Gurwitz et al. (2014)  United States  Quantitative | To evaluate the impact and effectiveness of an electronic health record (EHR)-based transitional care intervention with automated alerts to health care providers during the discharge of older adults | EHR-based transitional care intervention with automated alerts that notifies health providers of the following information: patient's recent discharge, new drugs added during the inpatient stay, warnings about drug-drug interactions, and recommendations for dose changes and laboratory monitoring of specific medications, alerts to primary care staff to schedule a post-hospitalization office visit |  |
| Hewner et al. (2014)  United States  Report | To report how informatics facilitated the shift of a care transition program from a single chronic disease management to a population-specific chronic disease management | Care transitions management intervention with population-based care and informatics (telephone outreach, screening, triage of individuals after hospital discharge to identify, predictive modelling, use of EHR, supportive services, postdischarge outreach calls) |  |
| Jonker et al. (2021a)  Netherlands  Quantitative | To examine and investigate the development of a new mobile application with information technology (IT)-supported integrated care management system for postoperative remote home monitoring of older adults after oncologic surgery | Mobile application and activity tracker with an IT-supported integrated care management system for postoperative remote home monitoring  Some patients used additional devices (e.g. thermometer, blood pressure monitor, weight scale) and completed electronic health questionnaires |  |
| Jonker et al. (2021b)  Netherlands  Quantitative | To understand how remote home monitoring aids older adults after hospital discharge following oncological surgery by measuring and monitoring physical activity, vital signs, and patient-reported symptoms. | The perioperative remote home monitoring intervention measures patients’ physical activity, vital signs, and patient-reported symptoms through commercially available monitoring devices and electronic questionnaires connected to a remote home monitoring system. This system comprises a tablet-based health application (self-management system) and a web-based application self-adaptive case management system ) |  |
| Jørgensen et al. (2021)  Denmark  Quantitative | To investigate the viability of a videoconferencing telerehabilitation intervention among older adults recently discharged from the hospital. | This 4-week intervention started within the first week of discharge. Patients received supervised telerehabilitation from an experienced physiotherapist for 3 days per week (>1 rest day between each session). The physiotherapist conducted home visits prior to the intervention to assist with equipment set up and explained the intervention format. After the first two sessions, the intervention was delivered in a group format. |  |
| Kim et al. (2021)  Canada  Quantitative | To analyze the data (sleep quality, physical activity, heart rate) obtained from the wearable device, in order to examine the progression of frailty after hospital discharge of older adults with critical illness | The wearable device was worn during and after hospital discharge for 4-weeks. This device collected the following information: step count, physical activity, sleep, and heart rate |  |
| Lafaro et al. (2020)  United States  Quantitative | To analyze the practicability of a personalized telehealth perioperative physical activity intervention for older adults who have had lung and gastrointestinal cancer surgery and their caregiver while examining the outcomes (i.e., trends, functional recovery, self-reported outcomes) of intervention before and after surgery | Personalized telehealth perioperative physical activity intervention (based on the chronic care self-management model), which includes a target goal for daily steps and recommendations on the use of lower extremity exercises tailored to the patient’s functional status before surgery |  |
| Liang et al. (2021)    Taiwan  Quantitative | To understand the practicability of a nurse-led tele-homecare intervention for older adults with multiple chronic conditions and high risk for hospital readmission | The intervention was a continuous telemonitoring program, where participants were provided with home visits and wireless transmission devices, such as a one-touch smartphone (allowed for communication with a 24hr call center, led by a nurse and reminders to check for vitals and take meds), blood pressure monitoring, medication dispenser, glucometer and a necklace emergency call button. These wireless devices were then transmitted to the 24-hr call center (led by eight senior nurses, with the assistance of physicians and technology engineers) and the healthcare record system | |
| Lindhardt et al. (2017)  Denmark  Mixed method study | To evaluate the effectiveness and feasibility of a technology-supported nutritional care program for older adults discharged from hospital | Technology-supported energy- and protein-enforced home-delivered meals for older patients discharged from hospital  The group received: 1) enriched meals delivered to participants’ homes 12 weeks after discharge, and 2) a tablet computer combining goal setting for intake with self-monitoring and feedback | |
| Luo et al. (2019)  China  Quantitative | To examine the usefulness of a nursing rehabilitation intervention delivered using WeChat to older adults after total hip arthroplasty | Nursing intervention via a chatting regarding the rehabilitation of patients after total hip arthroplasty | |
| Lyth et al. (2021)  Sweden  Quantitative | To understand the effects of a telemonitoring system (electronic Health Diary) on health care costs and the number of hospitalizations among older adults with chronic obstructive pulmonary disease and heart failure. | The eHealth Diary is a telemonitoring system with a digital pen paper-based Health Diary and a web-based application. The system allows patients to record their daily health status (e.g. symptoms, measurement values, and intake of medication) as a way for caregivers to become aware of early signs of patients’ deterioration | |
| Madigan et al. (2013)  United States  Quantitative | To assess the feasibility of telemonitoring on rehospitalizations and determine whether telemonitoring improved the health status of older patients receiving home care with heart failure, as well as multiple comorbid conditions and impaired functional status | Telemonitoring of blood pressure, pulse, oxygen saturation and weight that transmitted data to a home health care agency | |
| Markle-Reid et al. (2020)  Canada  Mixed-method study | To understand and analyze the effectiveness of an integrated transitional care stroke intervention for community-living older adults with stroke and multimorbidity patients while determining if health outcomes have improved | An integrated hospital-to-home transitional care stroke intervention, encompassing care coordination, home visiting, and IP case conferences, supported by a web-based application called My Stroke Team | |
| McCloskey et al. (2015)  Canada  Quantitative | To analyze the effectiveness of an interactive voice response telephone system for discharged (from geriatric rehabilitation unit) older patients and caregivers while assessing the recruitment and retention challenges of the study | Interactive voice response telephone system that transmitted monitoring data from participants’ home telephone to a secure hospital database | |
| McGillion et al. (2020)  Canada  Quantitative | To analyze the feasibility and acceptance of a Remote Automated Monitoring (RAM) and virtual hospital-to-home intervention for older adults following a cardiac and major vascular surgery | Postoperative RAM and Virtual Hospital-to-Home Care System Following Cardiac and Major Vascular Surgery  The Technology-Enabled Remote Monitoring and Self-Management—Vision for Patient Empowerment Remote Automated Monitoring and Virtual Care Intervention | |
| Mosca et al. (2020)    Italy  Quantitative | To evaluate the practicability of the GOAL (Games for Older Adults’ Active Life) telerehabilitation program for older adults with mild and vascular cognitive impairments. | The GOAL is a web application used to deliver an 8-week rehabilitation program comprising of a combination of cognitive, physical, and social weekly activities | |
| Pedone et al. (2015)  Italy  Quantitative | To evaluate the effectiveness of telemonitoring and telephone support on 6-month survival and hospital readmissions of older adults with heart failure | Telemonitoring system, which monitors vital parameters, including oxygen saturation, heart rate, and blood pressure and telephonic support from a geriatrician | |
| Piau et al. (2019)  France  Quantitative | To examine the performance and feasibility of a smartphone Chatbot application (semi-automated messaging) for older adults and to assess the health impacts of the intervention | A smartphone Chatbot application is a semi-automated messaging application (oncology nurses called patients every week after discharge from hospital under oncologist supervision) | |
| Oritz-Piña et al. (2021)  Spain  Quantitative | To understand the effects (patient-reported & performance-based) of a multidisciplinary telerehabilitation program on functional recovery of older adults with hip fracture by comparing a tele-rehabilitation program with a home-based in-person rehabilitation | A 12-week multidisciplinary telerehabilitation program (administered by family caregivers). The program comprised of two online components (three exercise sessions & two occupational therapy sessions) with pre-recorded instruction videos and written instructions on lower and upper body strengthening exercises, balance exercises, cardiovascular exercises, safest ways to perform activities of daily living, self-care activities, walking aids, and preventing falls. Furthermore, caregivers were able to request weekly videoconferences with therapists. | |
| Sabir et al. (2019)  United Kingdom  Quantitative | To examine the performance of using a web‐based intervention, called Connect with Pharmacy (CwP), as a tool to share discharge information with community pharmacies while also analyzing the impacts and outcomes of the intervention | CwP is a web‐based intervention designed to reduce the risk of hospital readmission among older adults | |
| Sorensen et al. (2021)  United States  Quantitative | To assess if and how a care transition intervention (an association of community-based health coaches and primary care-based pharmacists) results in improved medication management and a reduction in inpatient utilization among older adults following hospitalization | The care transition intervention comprised of an alliance between health coaches and primary care–based pharmacists. It involved in-hospital education and a follow-up after discharge (in-person and phone) provided by a health coach. The health coach also electronically transmitted health information to clinical pharmacists for medication reconciliation. The pharmacist used the electronic medical record to make recommendations, and with the approval of the patient’s primary care physician, the pharmacist made changes to medications. | |
| Villani et al. (2014)  Italy  Quantitative | To assess the clinical efficacy and cost of the telemonitoring and telecare system for older adults at high risk of rehospitalization | Clinical and psychological telemonitoring and telecare (consisting of a patient front- end, a medical front-end and a web-based system for assisting with clinical decisions) of high-risk heart failure patients | |
| Wade et al. (2012)  Australia  Quantitative | To investigate the acceptance of a telehealth intervention by older adults and carers enrolled in the Transition Care Program and determine whether telehealth acceptance predicts usage compliance | Tunstall® (Tunstall Healthcare, Yorkshire, UK) home monitoring system  Group 1 - non-telehealth controls and usual transition care  Groups 2 and 3 - transition care plus telehealth monitoring of blood pressure, heart rate, oxygen saturation levels, and bodyweight  Groups 4 and 5 - telehealth monitoring and used a pendant alarm | |
| Whitehouse et al. (2020)  United States  Quantitative | To determine the performance and effectiveness of diabetes self-management education and support (DSMES) sessions delivered through telehealth to older adults with type 2 diabetes mellitus after hospitalization | An in-person home visit was conducted within 24 to 48 hours of hospital discharge, followed by 4-week weekly one-on-one DSMES sessions provided virtually using an electronic tablet | |
