# Supplementary material for: Inclusion of Older Adults in Digital Health Technologies to Support Hospital-to-Home Transitions: Secondary Analysis of a Rapid Review and Equity-Informed Recommendations
Source: JMIR Aging. 2022 Apr 27;5(2):e35925. doi: 10.2196/35925 (PMC9096639; doi:10.2196/35925)
Supplement: Multimedia Appendix 3 [file aging_v5i2e35925_app3.docx]

| **First Author** | ***Population of interest (e.g. cardiac, stroke)*** | ***Ages targeted*** | ***Access to digital resources (e.g. device, Wi-Fi)?*** | ***Comfort with technology*** | ***Language limitations*** | **Recruitment strategy (e.g. Where are participants recruited from? Sampling strategy?)** | **Is there a justification for inclusion criteria (Yes (Y)/No (N)?) If Y, *describe justification provided*** | **Other details of inclusion criteria** | **Other details of exclusion criteria** |
| --- | --- | --- | --- | --- | --- | --- | --- | --- | --- |
| Agud et al. | Frail elderly/ very old acute (geriatric) participants | ≥75 years of age | Not Reported (NR) | NR | NR | 1300-bed university hospital (geriatric department) in Madrid, Spain | Y: Older adults discharged to supportive living may have nursing support to manage medication | Older adults who had >5 prescribed medications and were discharged alive between January 2013 and April 2014 | Older adults participants not discharged home |
| Amroze et al. | Older adults discharged (to home) from the primary hospital used by the medical group | >65 years of age | NR | NR | NR | Multispecialty medical group (217 physicians at 15 sites throughout Central Massachusetts) | N | Older adults who were members of local health plan with primary care providers within this medical group and discharged from August 26, 2010, to August 25, 2011 | NR |
| An et al. | Older adults scheduled to undergo total knee arthroplasty (TKA) | 65 to 75 years of age | NR | Familiarity with smartphone apps required | NR | Target sample size -54, recruited 60 participants from an orthopedic surgery rehabilitation hospital | N | Older adults scheduled bilateral TKA; efficient control of comorbid diseases; ability to independently walk >10 meters; average vision and hearing; can consent to participate in program; and no cognitive impairment and good communication ability | Older adults who had medical instability; inflammatory arthritis; scheduled TKA revision; history of knee surgery < 6 months; neurological disorders; cognitive impairment; and neurological damage to the lower extremity |
| Aziz et al. | Older adults scheduled for or have undergone elective surgical procedures | ≥60 years of age | No: Study team installed device in participants’ home | No: Participants were trained to wear the device | NR | NR | N | Older adults with postoperative length of hospital stay of > 7 days | NR |
| Backman et al. | Phase 1: Older adults who had hip fracture surgery, caregivers, clinicians (part of the Geriatric Rehabilitation Service)  Phase 2: Older adults being discharged from the Geriatric Rehabilitation Service after a hip fracture surgery | Phase 1/2: Older adults - ≥65 years of age;  Informal caregivers - ≥18 years of age | Phase 1: Loaner iPad (Apple Inc) provided to participants without personal mobile/ computer  Phase 2: Required access to a mobile or computer device | Phase 1: Training/support on device use was provided as needed | English speaking | Older adults being discharged from Geriatric Rehabilitation Service were invited to participate using convenience sampling | N | Older adults who were discharged home or to a community facility < 90 days | NR |
| Boeni et al. | Diabetes | NR | NR | NR | NR | Older adults were recruited from another pilot study | N | NR | NR |
| Chen et al. | Geriatric patients | NR | NR | NR | NR | Older adults admitted to the hospital’s acute aged care ward | N | Older adults admitted to the acute aged care ward between July and October 2008 | Older adults not admitted under a geriatrician or were transferred to another hospital ward before discharge |
| Choi et al. | Low-literate older adults recovering from hip replacement surgery | ≥ 65 years of age | NR | NR | Ability to communicate in English | Older adults were recruited from one rehabilitation hospital | Sample size of 15 was deemed appropriate due to data redundancy (i.e. no new insights after 13 participants) | Low-literate older adults - with marginal or inadequate health literacy (scoring ≤22 on the Short Test of Functional Health Literacy in Adults) | Older adults with cognitive impairments/conditions (screened using the Mini-Cog) or visual acuity below 20/60 with glasses |
| Gao et al. | Older adults with hip fracture | ≥65 years of age | WeChat | Older adult or >1 family member used WeChat | NR | Recruited older adults with a hip fracture who were treated in trauma and orthopedic surgery from February 1, 2020, to April 30, 2020 | N | Older adults who had low energy injuries; had internal fixation or unilateral total hip joint and femoral head replacement; and volunteered to participate | Older adults <65 years; had multiple or high energy fractures; ignored the use of chat software; did not use WeChat; had Alzheimer’s disease, consciousness disorder or mental illness; had a long-term bed rest or accompanied by other organic diseases that seriously affect functional exercise; were unwilling to participate in research; and lost contact |
| Guidetti et al. | Older adults with stroke | NR | NR | NR | Swedish speaking | Providers in an inpatient rehabilitation hospital unit and on primary care rehabilitation units recruited 4–5 participants with stroke from each unit in September 2017 | Recruited older adults regardless of time since stroke 🡪 stroke has long-term consequences on patient; hence, researchers thought it was beneficial to evaluate F@ce also after the initial rehabilitation period | Older adults with a stroke that were referred to one of the participating units and able to participate in an eight-week intervention | Older adults who did not start or continue rehabilitation in any of the participating units; had severe fatigue; severe aphasia; foot fracture; and depression |
| Gurwitz et al. | Older adults discharged home from the hospital | ≥65 years of age at the time of discharge | NR | NR | NR | Eligible older adults were identified daily using a computer program | Primary hospital used by the large multispecialty group practice from August 26, 2010 to August 25, 2011 | Older adults hospitalized for a surgical procedure with observation status and were being discharged from an inpatient facility after a non-psychiatric condition, discharged to the community | Older adults discharged to a hospice or a skilled nursing facility, rehabilitation facility, or long-term care setting |
| Hewner et al. | Chronically ill elders | NR | NR | NR | NR | NR | N | NR | NR |
| Jonker et al. | Older adults with oncologic conditions (hospital discharged after oncologic surgery) | ≥65 years of age | Internet access at home  Due to low recruitment rates, researchers provided interested participants who did not use a smartphone with a tablets  Participants with an old smartphone that was unsuitable to run the application were provided with a tablet | 7 individuals were eligible but declined participation due to digital illiteracy | Understanding of the Dutch language  Individuals who had insufficient understanding of the Dutch language were excluded | Eligible older adults were approached by a case manager before scheduled surgery in the surgery department at a tertiary center in the Netherlands | Y: Inclusion criteria were broadened during the study to those who had old smartphones and those without smartphones to address usability issues and low recruitment rates | Older adults with elective oncologic resection of a solid tumour, were able to provide written consent | Older adults with severe visual, hearing or cognitive impairment |
| Jonker et al. | Older adults undergoing cancer surgery | >65 years of age | Required internet access at home | Capable of using components of the remote home monitoring system | Dutch Language | NR | N | Older adults scheduled for surgical resection of a solid malignant tumour in the Department of Surgical Oncology or Department of Gynecological Oncology | Older adults with cancellation of surgery, emergency surgery, or perceived incapability to use components of the remote home monitoring system due to contact dermatitis, insufficient understanding of the Dutch language, or severe auditory, visual, cognitive, or ambulatory impairment |
| Jørgensen et al. | Geriatric participants with medical acute illness or hip fracture | >65 years of age | Did not require participants to install a particular network 🡪 a dongle was connected to the participant’s computer to obtain an internet connection  However, participant had able to obtain a stable network | The platform required basic computer skills and participants should be able to communicate through telephone or computer | Danish speaking | Participants admitted during the study period: 1st period from 15 March to 16 May 2018 & 2nd period from 1 September to 8 November 2018 | Inclusion criteria changed from 75 to 65 years 🡪 due to realizing the difficulties in using a computer and a computer mouse | Older adults were living in their own home, admitted to the Emergency Department and Department of Geriatrics of Aarhus University Hospital due to acute illness; aged 65 years or older; medical or hip-fracture patients; living in the municipality of Aarhus; dependent on a walking aid at discharge; and able to provide informed consent and see the computer screen | Older adults with a terminal illness; were unable to communicate via telephone or computer, were unable to walk independently with a walking aid; were unable to speak or understand Danish; suffered from dementia; had a Short Portable Mental Status Questionnaire score above 4; were unable to complete the Otago Exercises Programmewithout a great risk of falling or had suffered from acute stroke |
| Kim et al. | Older survivors of critical illness | ≥55 years of age | NR | NR | NR | A convenience sampling method was used to recruit patients with critical illness from Kingston General Hospital in Canada | Lived within the city or close by 🡪 to ensure the feasibility of attending the 4-week follow-up session | Older adults lived within the city or close by | Unavailable shared decision-makers to collect collateral history; had medical conditions that might have interfered with the proper use of the devices; diagnosis of primary neuromuscular pathology or atrial fibrillation; or used a wheelchair for mobility; had nonsinus rhythms; or had an expected survival <1 month |
| Lafaro et al. | Older adults with a diagnosis of lung or gastrointestinal (GI) cancers and are scheduled to undergo surgery; Primary Caregivers | Older adult: ≥ 65 years of age  Caregiver: age ≥ 21 years | NR | NR | Ability to read and understand English | Recruited from one National Cancer Institute–designated com- prehensive cancer center in Southern California over a 7- month period | N | Older adults who had a diagnosis of lung or GI cancer, were scheduled for surgery | NR |
| Liang et al. | Older adults with multiple chronic illnesses and high risk for readmission | >65 years of age | Data transmission from devices was wireless | Information technology engineer provided techno- logical support, including set up and illustrating instructions | NR | Recruited inpatients who were scheduled to receive home care after discharge, from a 400- bed regional hospital in Taiwan | N | Older adults at high risk for readmission, acuity of admission; comorbidity; visits to emergency department; LACE index of ≥7 (identifies patients that are at risk for readmission or death within thirty days of discharge); and willingness to participate | Older adults with terminal stage cancer; dementia with the inability to communicate; or admitted to a medical institution or nursing home facility |
| Lindhart et al. | Older adults identified with a nutritional risk score≥3 according to Nutritional Risk Screening (NRS) 2002 | >65 years of age | NR | NR | NR | Older adults admitted to 5 units of the Department of Internal Medicine in a large university hospital | Y: living within a radius of 20 km from the hospital 🡪 criteria justified by the fact that research assistants used bicycles to visit the participants | Older adults discharged to their own home and were living within a radius of 20 kilometers from the hospital | Older adults with food allergies or intolerance, vegetarians; terminally ill; unable to communicate and co-operate on the use of the tablet computer |
| Luo et al. | Older adults who underwent total hip arthroplasty (THA) | > 65 years of age | Able to be contacted via telephone after discharge | Able to personally use WeChat or have family members that could send the WeChat content to the patient in a timely manner | NR | Participants were recruited from the Honghui Hospital from January 2013 to October 2015 | N | Older adults with THA; cognitive and communication function within normal range were willing to participate in the study; could provide written informed consent; and at minimum junior high school-level education | Older adults who had mental disorders or serious chronic diseases (heart, lung or brain); could not independently perform self-care; or had participated in a similar study |
| Lyth et al. | Older adults with chronic obstructive pulmonary disease (COPD) or HF | >65 years of age | NR | NR | Swedish language | Participants were recruited until the required study number was reached (n = 65 in each group) according to sample size calculation | N | Older adults who visited the emergency department at Linkoping University Hospital due to exacerbation for either COPD or HF with a history of >two inpatient episodes < 12 months  Participants with inpatient care at other departments at Linkoping University Hospital and patients in primary care if their HF or COPD condition worsened | Older adults with dementia; other cognitive impairment; or psychotic illness; unable to understand Swedish; severe hearing loss; underwent or scheduled surgery <six months;or other life-threatening illness |
| Madigan et al. | Older adults referred to home health care (HHC) agencies with HF | NR | Working phone line needed | NR | NR | Participants referred to a participating HHC agency by the discharging hospital  Randomized | Participants with HF residing in the community with multiple comorbid conditions and impaired functional status and were referred for HHC as part of their hospital discharge plan 🡪 justified by the fact that the researchers wanted to mimic a real situation (i.e. consider the type of patients who would receive HHC in the community) | Older adults with HF (i.e. HF diagnosis (primary or secondary); New York Heart Association (NYHA) class II-IV; reside in community; had multiple comorbid conditions and impaired functional status | Older adults who were unable to stand on a scale; weighed >500 pounds; had a hearing or cognitive impairments; no working phone or an unstable health condition; dependent on oxygen; active cancer, AIDS, on dialysis or uncorrected thyroid disease |
| Markle-Reid et al. | Community living older adults with stroke and multimorbidity | >55 years of age | NR | NR | Competent in English or with an interpreter available | Potential and eligible participants identified and approached in person prior to their discharge or by telephone, by a recruiter, to obtain verbal consent of participation  Participants were all allocated the intervention | Y: older adults with multimorbidity are often excluded from stroke rehabilitation studies | Older adults who had multimorbidity; were newly dis- charged from hospital and referred to outpatient stroke rehabilitation services; hospitalized for a stroke within >1year; >2 chronic conditions; were not living in a long-term care home; mentally competent or had a substitute decision-maker; resided within geographical boundaries of the outpatient stroke clinic | NR |
| McCloskey et al. | Older adults who are transferred to geriatric rehabilitation unit after resolution of an acute medical or surgical condition that required hospitalization | NR | A standard push-button telephone was needed, but no special equipment was required | Series of 1:1 instructional sessions (i.e. demonstrations of how to call into the system, an overview of questions that would be asked, and how to answer each one) for using the device provided to participants  Sessions allowed participants to practice, ask questions and educational materials with detailed written and graphical instructions on how to use the system were provided to participants  Manual dexterity (to enter data on telephone)  Older adultshad to be able to independently operate the interactive voice response telephone system without assistance or supervision | NR | Study participants were recruited from a geriatric rehabilitation unit within a hospital located in New Brunswick, Canada | Daily contact with an informal caregiver required 🡪 to understand if/to what extent the system supported them | Older adults who were scheduled for discharge to a private home in the community; normal vision and hearing; require some assistance with activities of daily living or instrumental activities of daily living at the time of discharge; and have a family caregiver who would have daily contact with them after they returned home | Older adults with cognitive impairments |
| McGillion et al. | Participants included surgical ward nurses and older adults recovering from cardiac or major vascular surgery | >65 years of age | NR | Patients received individualized training sessions (1-hour long) on the use of technology | Able to read, speak, and understand English | Brief presentations at staff meetings and nursing rounds, emails sent by ward managers were used to recruit nurse participants from the surgical ward | N | Ambulatory older adults recovering from a cardiac or major vascular surgery | Older adults with symptoms/signs of postoperative delirium |
| Mosca et al. | Older adults with Mild Cognitive Impairment (MCI)& Vascular Cognitive Impairment (VCI) | between 65 and 80 years of age | NR | NR | Italian language as mother tongue | Recruited from the Memory Clinic of University Hospital of Careggi, (Florence, Italy) and the Don Carlo Gnocchi Foundation (Florence, Italy) | N | Older adults who had a MCI or VCI; “Mini Mental State Examination score >24”*; school attendance >3 years; right- handed; normal or corrected visual and auditory acuity; preserved physical mobility or manual dexterity  *Reported by paper | Older adults with mild dementia; intellectual deficiency; alcoholism or toxicomania; use of psychotropic medication known to impair cognition; presence or history of severe psychiatric disorders; presence or history of stroke; presence or history of a neurological disorder; and general anesthesia in >6 months |
| Pedone et al. | Older adults with HF | >65 years of age | NR | NR | NR | Randomized. Participants were recruited from the geriatric acute care ward of a teaching hospital, and the outpatient clinic of the same hospital | N | Older adults with a diagnosis of HF | Older adults with severe cognitive impairment |
| Piau et al. | Older adults with cancer in any hospital care unit; undergoing chemotherapy after a cancer diagnosis | >65 years of age | NR | NR | NR | Recruitment following a pragmatic approach to reflect real practices | N | Older adults undergoing chemotherapy for cancer | No exclusion criteria |
| Oritz-Piña et al. | Older adults admitted with hip fracture | >65 years of age | Required internet access and/or a family caregiver with access | NR | NR | Recruitment took place at the University Hospital of Granada between January 2017 and July 2018 | N | Older adults who had hip fracture surgery; had a high (self-reported) pre-fracture functional level the week before the fracture (Functional Independence Measure index > 90 points); could weight- bear at 48 hours after surgery; and community-dwelling (in own home or with a relative) after hospitalization | Older adults with presence of severe cognitive impairment (Mini-mental State Examination score lower than 24 points); terminal disease (<6 month survival); or post-surgery complications |
| Sabir et al. | Phase 1 examined older adults’ use of a compliance aid, which would be tested at a program-level | >65 years of age | NR | NR | NR | NR | N | Older adults discharged home from the hospital. If patient lacked capacity, the pharmacy staff member could enroll the patient in the study if they thought it was in the patients’ best interest | Older adults with elective admissions |
| Sorensen et al. | Hospitalized older adults for a nonelective reason  Older adults | ≥65 years of age | NR | NR | Primary language among participants was English | Older adults were recruited from the general medicine inpatient wards at University of California, Los Angeles Health System’s (UCLA) Ronald Reagan Medical Center | Risk factors used for inclusion derived from a root cause analysis conducted by UCLA Health researchers that aimed to identify risk factors for readmission | Older adults hospitalized for a nonelective reason; had an assigned UCLA pri- mary care provider; had Medicare fee-for-service insurance coverage; discharged home after hospitalization; had >2 risk factors hospital readmission <30 days and/or two or more admissions >12 months; hospital stay > 10 days; >eight outpatient pre- scription medications; depression as a secondary diagnosis; mild cognitive impairment; >two chronic conditions; and limited caregiver support | Older adults who were homeless, sent to hospice on the day of discharge; in an observation unit; had a primary admission diagnosis of mental disease and/or substance abuse; or were admitted for scheduled or recurring chemotherapy, immunotherapy, radiation therapy, rehabilitation, or dialysis |
| Villani et al. | Older adults with chronic HF leaving hospital after being treated for clinical instability | > 70 years of age | NR | Participants learned to use a handheld device and kept in touch daily with the monitoring centre | NR | NR | N | Older adults with NYHA class III/IV during hospital stay; left ventricular systolic dysfunction (ejection fraction 40%); and high risk of early re-hospitalization at discharge | Older adults with social cognitive problems (e.g. alcohol or drug abuse; psychiatric disease); inability; or refusing to learn how to use the device, life expectancy of <12 months |
| Wade et al. | Frail older adults from a community- based Transition Care Program  Carers of participating older adults | NR | Phone line connected to the house, or power source in a suitable location in the house or a place install the equipment, which would not be a hazard to the participant or potentially result in damage to the equipment is required  Participants in groups 2–5 provided with ahome monitoring system | NR | Sufficient English | Stratified random sample  Recruited participants over a period of 14 months to October 2010. | N | Older adults transitioning home from the hospital; had a chronic disease; and were at risk of being admitted into residential care. If a participant lacked capacity but had someone to assist them with the equipment and the readings, they were included | Older adults lacking capacity to complete study procedures or if they had a cognitive impairment and had no carer who was willing to participate; no phone line or suitable power source to install equipment; were using another telehealth product |
| Whitehouse et al. | Hospitalized older adults with type 2 diabetes | ≥55 years of age | NR | Prior technology use ofactual sample: 16 (80%) | English speaking | Screening and recruitment done by an advanced practice nurse/certified diabetes educator who approached potential participants during hospital admission | NR | Older adults with an admission to a medical or surgical unit for any reason; were cognitively intact; resided within 30-mile radius of the study site | Older adults with cognitive impairments |
