# Supplementary material for: Inclusion of Older Adults in Digital Health Technologies to Support Hospital-to-Home Transitions: Secondary Analysis of a Rapid Review and Equity-Informed Recommendations
Source: JMIR Aging. 2022 Apr 27;5(2):e35925. doi: 10.2196/35925 (PMC9096639; doi:10.2196/35925)
Supplement: Multimedia Appendix 4 [file aging_v5i2e35925_app4.docx]

**Multimedia Appendix 4: Details of the participants (ie, actual sample)**

**Table 3**

| **First Author** | **Total sample size (n=)** | ***Age*** | ***Sex, n (%)*** | ***Ethnicity, n (%)*** | **Any limitations reported relating to the target sample (Yes (Y)/No (N))? If Y, please list.** | **Any limitations reported relating to the actual sample (Y/N)? If Y, please list.** | **Other details of sample** |
| --- | --- | --- | --- | --- | --- | --- | --- |
| Agud et al. | 118 | Mean 87 years (SD 6) | Male: 61 (52%)  Female: 57 (48%) | Not Reported (NR) | N | Medication reconciliation could not be performed for 100% of the discharged patients | 2054 medications reviewed & reconciled (17.4 medications per patient)  Included “very old acute patients” |
| Amroze et al. | 713 | Patients:  65-74 years: n=255 (32%);  75-84 years: n=349 (44%);  ≥85 years: n=195 (24%) | Patient characteristics  Male: 381 (48%)  Female: 418 (52%) | NR | N | N | NR |
| An et al. | 60 | Preoperative telerehabilitation groupMean 71 years (SD 3)  Patient Education Group Mean 70 (SD 2)  **Control (usual care)** Mean 70 years (SD3) | Female: 60 (100%) | NR | N | Small sample size and only females | NR |
| Aziz et al. | 5 | Mean 74 years (SD 8) | Male: 3 (60%)  Female: 2 (40%) | NR | N | Participant 1 had limited exercise tolerance; left house only twice a week due to his shortness of breath which limited his walking to about 366 m (400 yards) at a time | Comorbidity, exercise tolerance, living situation (most lived alone), operation details, postoperative complications |
| Backman et al. | 48 | Phase 2: Mean 81 years (range 67-96) | Phase 2:  Male: 11 (37%) Female: 19 (63%) | Phase 2 - Caucasian: 28 (93%)  Other: 2 (7%) | N | N | Highest level of education completed (most were highschool educated), relationship status (most were widowed) and living situation (most lived alone) |
| Boeni et al. | 1 | 65 years | Male: 1 (100%) | NR | N | N | 65-year-old man, hospitalized at a large Swiss university hospital, from 28 January to 18 February 2013, for sepsis by Staphylococcus aureus |
| Chen et al. | 168 | Mean 85 years (range 63–100) | Male: 58 (34%) Female: 110 (66%) | NR | N | Y-Limited to geriatric patients from an acute aged care ward | discharge location |
| Choi et al. | 15 | Mean 68 years (SD 5) | Male: 6 (40%) Female: 9 (60%) | White: 9 (60%); African American: 3 (20%); Asian/Pacific Islander: 1 (7%); Hispanic: 2 (13%)  Participants were racially diverse | N | N | Education (most had high school or equivalent)  Mean scores on Short- Test of Functional Health Literacy in Adults indicated sample had marginal or inadequate health literacy |
| Gao et al. | 80 | Intervention: Mean – 78 years (SD 6)  Control: Mean 78years (SD6) | Male: 30 (38%)  Female: 50 (62%) | NR | N | N | All participant tested negative for the virus.  60 days after discharge, there were six deaths in the control group and one in the observation group; 15 participants in the control group had complications, while only six did in the observation group.  Main complications were deep vein thrombosis, urinary tract infection, heart failure, incision infection, lung infection, dislocation of the prosthesis, and fracture around the prosthesis. |
| Guidetti et al. | 10 | Mean 65 years (SD 12) | Male: 5 (50%) Female 5 (50%) | NR | N | Small sample size  No control group | All participants had suffered a mild stroke. Most participants lived alone and worked pre-stroke. Cognitive test score of three participants indicated a cognitive impairment.  Five had a recent stroke (≤1 month before inclusion), and five had suffered a chronic stroke (10 to 32 months before inclusion) |
| Gurwitz et al. | 3661 | Intervention: Mean 79 years (SD 7)  Comparison: Mean 79 years (SD 7) | Intervention Male: 880 (47%)  Female: 990 (53%)  Comparison  Male: 860 (48%) Female: 931(52%) | NR | N | N | Hospitalizations, office visits, number of different providers, prescriptions filled, lab tests performed in the past year, and comorbidity (diabetes most common) |
| Hewner et al. | NR | NR | NR | NR | N | N | Chronically ill elders |
| Jonker et al (a). | 47 | Mean 72 years (SD 5) | Male: 31 (66%) Female: 16 (34%) | Dutch: 47 (100%) | N | N | Living situation (most resided with others), highest level of education (most had secondary school-level), current employment status (11% currently employed), Charlson comorbidity index (median 4; range 2-6), frailty, functional and nutritional status, physical status and activity level |
| Jonker et al (b). | 58 | Mean 72 years (SD 5) | Male: 38 (66%) Female: 20 (34%) | NR | N | N | Reason for surgery (gastrointestinal malignancy most common), Charlson Comorbidity Index (median 3, range 2-6.8), frailty, functional, nutritional and psychological status |
| Jørgensen et al. | 2 (333 referred, 33 eligible, 9 agreed but only 2 enrolled) | Mean age of 33 eligible participant: 82 years (SD 8) | Male: 8 (24%)  Female: 25 (76%) | N | Participants were fatigued following hospitalisation and unable to cope with new initiative immediately 🡪 reason for declining study participation / dropping out | Some received home care for the first time, which meant many new people visiting their home, sometimes multiple times a day and some did not feel familiar enough with using a computer 🡪 reason for declining study participation /dropping out | NR |
| Kim et al. | 12 | Mean age 67 (SD 7) | Male: 5 (42%)  Female: 7 (58%) | NR | Patients from Kingston General Hospital in Canada | N | Length of hospital stay, Acute Physiology and Chronic Health Evaluation II score, Glasgow Coma Scale score, Charlson Comorbidity Index score |
| Lafaro et al. | 68 (34 patient and caregiver dyads) | Median age (n=34 patients) 73 years (range 66-84)  Median age (n=34 caregivers)  69 years (range 45-85) | Patients:  Male: 20 (59%)  Female: 14 (41%)  Caregivers:  Male: 14 (41%)  Female: 20 (59%) | Patients  White: 28 (74%)  Others 9 (26%)  Caregivers: White: 25 (74%)  Other: 9 (26%) | Heterogenous sample 🡪 composed of GI and lung cancer patients undergoing a wide range of surgical procedures with different risk and complication profiles | A small sample size was chosen with the intent to determine proof-of-concept | Highest education level (most patients had a college/graduate education), marital status (most patients were married), living situation (most patients did not live alone), employment status (most patients were disabled/retired; caregivers were mostly employed) |
| Liang et al. | 200 | Patients: Mean 81 years (*SD* 7)  Caregivers: Mean 52 years (*SD*  12) | Patients:  Male: 84 (42%)  Female:116 (58%)  Caregivers: Female – (68%) | NR | Participants from a single teaching hospital | Small sample size | Most participants were widowed and living with children. Most caregivers were the children of the participant  The mean LACE score was 9.81 (SD = 2.11).  Participants did not meet the reimbursed home care services criteria |
| Lindhart et al. | 25 (completed study) | Intervention: Mean 79 years (SD 8)  Control: Mean 80 years (SD 7) | Intervention:  Male: 2 (22%)  Female: 7 (77.9%)  Control:  Male: 8 (50%)  Female: 8(50%) | NR | N | Y: Small and biased sample size due to the low acceptability of intervention (particularly among married people) and high dropout rate  Intervention sample was primarily older women who lived alone | Marital status (most were unmarried), length of education (mean 8 years (SD 2) in control and 8 (SD 1) in intervention group) |
| Luo et al. | 232 | Group A: Mean 74 years (SD 13)  Group B: Mean 73 years (SD 13) | Group A:  Male: 35 (30%)  Female: 79 (69%)  Group B:  Male: 36 (30%)  Female: 82 (70%) | NR | Limited to elderly individuals who had capacity to use WeChat independently and had a higher degree of education or those who are dependent on family members for health education via WeChat | N | Living status (approximately half lived alone), dependent on help (31% were dependent), monthly income (most earned between 200-500 US) |
| Lyth et al. | 94 | Heart failure (HF) group: Median 84 years (range 65 to 100)  chronic obstructive pulmonary disease (COPD) group: Median 74 years (range 65 to 86) | HF group:  Male: 29 (50%)  Female: 29 (50%)  COPD group:  Male: 14 (39%)  Female –22 (61%) | NR | N | Unable to recruit the number of participants estimated from the sample-size calculation within the inclusion period of three years. | The majority (97%) of participants in the HF group were classified as NYHA 3B-4 of the New York Heart Association Functional Classification.  The majority (72%) of patients in the COPD group had very severe disease (stage IV) according to the GOLD-staging system |
| Madigan et al. | 99 | Intervention: mean 75 years (SD 12)  Usual care: mean 75 years (SD 11) | Usual care:  Male: 18 (45%)  Female: 27 (60%)  Intervention:  Male: 14 (26%)  Female: 40 (74%); | Usual care:  African American: 12 (26.7%)  Intervention:  African American: 9 (16.7%)  Patients were mostly Caucasian | Among the target sample that was referred to home care, most of them had a severe chronic illness and were disables | Actual sample was hesitant to be in the usual care group and preferred telemonitoring due to its potential benefits | Most patients that refused participation had severe chronic illness/disability  Reported health status, morbidities (diabetes, AFib most common), stroke etiology and severity, and medication |
| Markle-Reid et al. | 30 | Mean 72 years | Male: 16 (53%)  Female: 14 (47%) | NR | Participants were from only one hospital-based outpatient program | Sample size was small (but done intentionally)  No comparison group (in the one-group pretest/post-test design) among the actual sample | Most living with a family member, married, and had annual income <$40,000 CAD, time since stroke, history of stroke, number of prescription medications, falls in last year, depressive and anxiety symptoms, cognitive impairment status (almost all had cognitive scores indicating “they were mentally competent”) |
| McCloskey et al. | 5 | NR | NR | NR | Patients were recruited from a single rehabilitation unit  The need for patients to have a family caregiver who is willing to participate limited the number of patients in study  Target sample of frail older adults 🡪 since they have been hospitalized for a long period of time, they may be experiencing high levels of stress with participation in study | 2 participants withdrew before the first scheduled visit and the other three participants withdrew within 1 month  Reasons for withdrawal were unknown  The study was not completed by anyone | NR |
| McGillion et al. | 37 (26 nurses and 11 patients) | Mean age NR  (all patients were >65 years) | Nurses:  Male: 4 (15%) Female: 22 (85%)  Patients: Male: 7 (64%)  Female: 4 (36%) | Nurses: White: 21 (81%)  African decent: 4 (15%)  Asian: 1 (4%)  Patients: White: 11 (100%) | N | Homogenous sample  Only 1 patient had undergone vascular surgery, while the rest of the participants had all undergone cardiac surgery | Marital status (most were married), level of education (most had some high school education) and employment status (most were retired) |
| Mosca et al. | 61 | Completed: Mean age - 74 ± 4 | Completed:  Male: 19 (73%)  Female: 12 (34%) | NR | N | N | Years of education (mean 10 SD 5), cognitive impairment (55% had an impairment), functional status |
| Pedone et al. | 96 | Intervention: 80 years (SD 7)  Control: mean 80 years (SD 8) | Intervention: Male: 22 (47%)  Female: 25 (53%)  Control:  Male: 13 (30%)  Female: 30 (70%) | NR | N | Small sample size  Characteristics among the control and intervention groups were imbalanced  Sample exhibited high prevalence of physical impairment | Include functional status, illness rating |
| Piau et al. | 9 | Mean 83 years (SD 2) | Male: 5 (56%)  Female: 4 (44%) | NR | N | Small sample size 🡪 unable to make conclusions (i.e. demographic or health participant’s profile regarding adherence/compliance behaviours) | Cognitive status scores, functional status,, number of drugs, number of hospital admissions in previous years and condition-specific health measures) |
| Oritz-Piña et al. | 71 | Control group: Mean age - 80 ± 5  Intervention group: Mean age: 76 ± 6 | Control:  Male: 9 (26%) Female: 27 (74%)  Intervention: Male: 8 (29%); Female: 20 (71%) | NR | N | Small sample size | Body Mass Index, functional outcomes |
| Sabir et al. | 627 | Mean 82 years  (SD 7) | NR | NR | N | N | Participants under 65 years were removed from the cohort during analysis  Incomplete, not “actioned”, unclaimable and rejected referrals were all excluded from the analysis |
| Sorensen et al. | 2964  (2470 control and 494 intervention) | Control group: Mean 83 years  Intervention group: Mean 83 years | Control:  Male: 929 (38%)  Female: 1541 (63%)  Intervention:  Male: 187 (38%)  Female: 307 (62%) | Control: *Race:* White: 1674 (68%)  Black: 332 (13%)  Asian: 173 (7%)  Other/Unknown: 291 (12%)  *Ethnicity:* Hispanic: 319 (13%)  Intervention:  *Race:* White: 326 (66%)  Black: 75 (15%)  Asian: 37 (8%)  Other/Unknown: 56 (11%)  *Ethnicity:* Hispanic: 69 (14%) | Participants were only from a single urban health system | Uncertainty of whether a patient was readmitted to a hospital outside of UCLA Health | Primary language English (86% in intervention and 83% in control), partnership status (44% in intervention and 45% in control had a partner), comorbidities (hypertension most common), number of medications, hospital/emergency department visits, length of stay |
| Villani et al. | 80 | Mean 72 years (SD 3) | Males: 59 Females: 22  *sum>80 (as reported in the manuscript) | NR | N | N | Smoking status, psychological measures and condition-specific health measures (e.g. New York Heart Association Class) |
| Wade et al. | 90 (61 clients, 29 carers) | Intervention group (n=32)  Mean (age of patients): 80 years (range 63-95) | Intervention group  Patients:  Male: 12 (38%) Female: 20 (62%)    Carers:  Female: 55% Male: 45% | NR | N | N | Patient: 15 had a carer (47%) and 17 did not (53%)  Carers:  Daughters: 38%; Husband: 25%; Son: 20%; Wife: 17%  68% - same residence (with participant); 32% - separate residence  90% caring for participants who were able to make their own health choices and 10% cared for participants who did could not  50 - who were already using another form or brand of telehealth equipment and wanted to continue to do so were excluded |
| Whitehouse et al. | 20 | Mean 66 years (SD 9) | Male: 9 (45%) Female: 11 (55%) | Black 15 (75%)  White 4 (20%)  Asian 1 (5%) | Recruited participants from one academic medical center in Philadelphia, Pennsylvania | Small sample size  No comparison group among the actual sample | Marital status (most single), level of education (high school and some college most common), employment status most retired), prior technology use (80% previously used technology), prior diabetes self-management education (40%) and support program (20%) |
