# Supplementary material for: Inclusion of Older Adults in Digital Health Technologies to Support Hospital-to-Home Transitions: Secondary Analysis of a Rapid Review and Equity-Informed Recommendations
Source: JMIR Aging. 2022 Apr 27;5(2):e35925. doi: 10.2196/35925 (PMC9096639; doi:10.2196/35925)
Supplement: Multimedia Appendix 5 [file aging_v5i2e35925_app5.docx]

| **Equity variable** | **Target sample** | **Actual sample** |
| --- | --- | --- |
| *Age* | How are older adults defined? If limiting maximum age, provide rationale. | Report age of participants and describe which age groups are underrepresented within sample. |
| *Race, ethnicity, culture* | Consider strategies to recruit a racial, ethnic, and culturally diverse sample. | Report race, ethnicity and culture of sample. |
| *Sex and gender diversity* | Consider strategies to maximize sex and gender diversity. | Capture and describe sex and gender of sample. |
| *Sexual orientation* | Consider strategies to maximize representation of people with different sexual orientations. | Capture and describe sexual orientation of sample. |
| *Education* | Consider strategies to maximize representation of people with different sexual orientations. | Capture and describe sexual orientation of sample. |
| *Disability* | Consider how to design technology to support the needs of people with various disabilities and health and functional statuses. | Capture and describe disability-related factors and consider which groups may be excluded from the sample. |
| *Language* | Consider translation of study material to enable participation of non-English speakers. | Capture and describe the primary language of sample. |
| *Technology access and comfort* | Consider strategies to include individuals without technology access (e.g. providing loaner device) and with low comfort with technology (e.g. additional training). | Capture and describe participants’ technology access and comfort with technology. |

**Multimedia Appendix 5: List of research implications**

**Table 4. Considerations of equity-variables within target and actual samples of digital health interventions**
